# The Influence of Decision Making on Social Inclusion of Persons with Disabilities: A Case Study of Khyber Pakhtunkhwa

**DOI:** 10.3390/ijerph19020858

**Published:** 2022-01-13

**Authors:** Shakeel Ahmad, Mazhar Islam, Muhammad Zada, Afraseyab Khattak, Rezwan Ullah, Heesup Han, Antonio Ariza-Montes, Luis Araya-Castillo

**Affiliations:** 1 Department of Social Work and Sociology, Kohat University of Science and Technology (KUST), Kohat 26000, Pakistan; dr.shakeel@kust.edu.pk (S.A.); mazharislam@kust.edu.pk (M.I.); 2 Business School, Henan University, Kaifeng 475000, China; 3 The University of Agriculture, Peshawar 25000, Pakistan; Siab4@aup.edu.pk; 4 School of Management and Economics, Beijing Institute of Technology, Beijing 100081, China; rezwanullah1990@yahoo.com; 5 College of Hospitality and Tourism Management, Sejong University, Seoul 05006, Korea; 6 Social Matters Research Group, Universidad Loyola Andalucía, C/Escritor Castilla Aguayo, 4, 14004 Córdoba, Spain; ariza@uloyola.es; 7 Facultad de Economía y Negocios, Universidad Andrés Bello, Santiago de Chile 7591538, Chile; luis.araya@unab.cl

**Keywords:** social inclusion, person with disabilities (PWDs), decision making, gender, level of disability, monthly family income

## Abstract

Decision making is an essentially social process adopted by individuals or groups to identify and choose the best choice among several alternatives. Decision-making choices are influenced by the preferences, values, and beliefs of the individuals or groups involved in the decision-making process. This study was conducted to analyze the social inclusion of Persons with Disabilities (PWDs) in the context of their participation in decision making. The study area consisted of 8028 PWDs registered with the government of Pakistan, from which a sample of 488 PWDs was selected through a multistage stratified random sampling technique. These PWDs included physically disabled, blind, crippled, and deaf persons; however, the data were collected from those who were able to be interviewed. Chi-square and Kendall’s Tau-b tests were used to determine the strength, level, and direction of association among variables. At the multivariate level, the study found a spurious relation between social inclusion and participation in decision making when controlling for gender, monthly family income, and level of disability of the PWDs. The results highlighted that participation in decision making improved the social inclusion of male and moderately disabled PWDs more positively. However, participation in decision making was a universal feature explaining the social inclusion of PWDs irrespective of their monthly family income. The logistic regression model explained that the social inclusion of PWDs was more likely to occur when PWDs were married, had high monthly family income (PKR 16,500 and above), belonged to a joint family, and actively participated in decision making. The study recommends that ensuring the participation of PWDs in decision making in family, community, school, and other relevant institutions ultimately enhances their social inclusion.

## 1. Introduction

Economic well-being has been considered a significant, sometimes sole contributor in molding the overall human life. Therefore, all past efforts for improving human life are centered on the issue of economic growth and increase in income only. However, economic ways of thinking to solve human miseries have failed to cater to the multifaceted and complex nature of disadvantages that shape human miseries. Hence, a need for a new approach is felt that should consider, besides economic deprivation, the factors of participation in the decision-making process that enable humans to lead a dignified life without discrimination. This holistic approach is better explained by social inclusion [1,2]. Social inclusion does not rely on the static economic factors of deprivation, like income and employment, alone; instead, it refers to the dynamic process to solve the overall disadvantage condition in a specific economic and social context. Through these factors, catering for social inclusion is meant to increase overall human prosperity for dignified and prosperous human life. The social inclusion process also includes encouraging marginalized segments of society to develop their skills and competencies to lead a dignified, decent, successful, and healthy life, and effectively participate in the decision-making process at the familial and societal level [3,4,5].

The social inclusion process takes care of all the individuals and groups that are either unable to participate in social life or shut out by society not to participate through their denial. The inability of individuals includes their physical, psychological, and mental incapability of participating in mainstream society [6]. Persons with disabilities are more likely to be shut out by society and are treated with discrimination concerning their social relations and participation in physical and customary activities, as well as sometimes being unable to make decisions about their private life. Thus, a fissure is created between PWDs and abled persons, comprising a major reason for societal disintegration [7,8,9]. In this way, a threat has arisen to disrupt the societal order, compelling legislators to devise laws and policies to reintegrate the marginalized and excluded groups into the mainstream [10,11,12]. Therefore, social inclusion is the state of having the resources and opportunities to participate in social, economic, and cultural life, and to enjoy the standard life considered normal in the society in which we live [13].

Social inclusion and participation put into effect a state where all individuals are free to enjoy their rights and are authorized to make personal, familial, and communal decisions [14]. Due to a lack of knowledge about the needs of PWDs in policies and services, they are unable to participate in familial and communal decisions and are excluded from economic, political, social, and cultural communities [4,15,16]. PWDs have fewer chances to make decisions about themselves than abled persons. Making decisions is vital to a person’s sovereignty and their spirit of personhood. It is a key component for enabling individuals to exercise their authority, have control over their lives, and interact with others in society. Those with an inability to make decisions about themselves are considered non-persons before the law and are socially excluded [16,17]. In these kinds of circumstances, others decide on behalf of disabled persons, weakening the decision-making ability of PWDs and maintaining low self-determination [18,19].

The CRPD demands a paradigm shift in the disability sphere, moving from a substitute decision-making model to a supported human rights-based model [20]. Article 12 of the CRDP states that PWDs must have equal recognition before the law [21]. Internationally, the law recognizes that PWDs may exercise their rights and are authorized to take decisions at all levels, e.g., with respect to access to education, employment opportunities, housing, health services, family relationship, property, and finances [12,20]. In the same way, countries have devised various policies and established public offices that facilitate PWDs in exercising their rights [21]. PWDs are excluded from the vital aspect of their lives and shunned to decide on personal, familial, and community levels. In addition, sometimes they have less capacity and are incapable of taking such decisions [22,23]. Generally, PWDs disclose that they have been avoided by able-bodied people during interactions due to their disabilities and have remained excluded from active participation in decision making and cultural life [20,24]. It has been pointed out that PWDs are ignored when making decisions at the family level; they are not consulted in family meetings and dialogues and their opinion is never considered in family discussions and decisions [23,25,26]. This exclusion from the vital aspect of their life results in low self-determination and develops incompetency among PWDs [2]. Such feelings lead to a low level of participation in decision-making processes [27]. For the social inclusion of PWDs in mainstream society, various approaches have been adopted by countries, and these have been modified from time to time. These approaches include the charity model, the medical model, the social model, and the rights-based model. The rights-based model is the most recent, and focuses on the equal participation of PWDs in all aspects of their lives, becoming productive members of society.

Rehabilitation of PWDs is a problem faced by both developed and developing nations. However, developing nations are shouldering the greater share of this problem. The United Nations took notice of this problem when the UN sectary general highlighted the issue in 1984 by stating that 20–25% of the population of developing countries were affected by disability. The report also stated that 350–500 million Persons with Disabilities (PWDs) lived in areas with inappropriate and insufficient services required for these persons (UN, 1984). The population of PWDs jumped from 10% to 15% of the world population from 1970 to 2010 [28]. The United Nations Convention on the Rights of Persons with Disabilities [20] defines disability as “persons with disabilities include those who have long-term physical, mental, intellectual or sensory impairment which in interaction with various barriers may hinder their full and effective participation in society”. The World Report on Disability [28] estimated that more than a billion people live with some form of disability, representing about 15% of the world’s population. In Pakistan, a sharp decline of 80% in the total population of PWDs from 1998 to 2017 was recorded in the 2017 census report. Only 0.48% of the national population was categorized as disabled. These disabled persons include the blind, the deaf/mute, the insane, the intellectually disabled, and the physically disabled, including those who are crippled, those having multiple disabilities, and others [29]. In this research study, blind and physically disabled people, including those who were crippled and those who were able to participate in the study as interviewees, were the main focus.

### 1.1. Types of Disability

There are various types of disability reported globally. Some of the common types of disability include:

- Deaf or hard of hearing: those who are deaf or hard of hearing need to use some type of equipment for hearing [30].
- Physical disability: people with physical disabilities are those whose physical differences are visible, as evidenced by a missing limb, atypical gait, or the use of a wheelchair. In addition, with a physical disability, the person’s physical functioning, i.e., mobility, dexterity, and stamina, is affected [30].
- Mental health conditions: this is a general group of illnesses that affect the person’s brain or mind. In addition, such mental problems affect how a person thinks, feels, acts, etc. [31].
- Vision impairment: people with vision impairment are those who are fully blind or partially blind (partial vision) [31].

In this research study, persons with vision impairment and physical disability were targeted to measure their participation in decision-making processes and their social inclusion.

### 1.2. Main Objectives of the Study

- To examine the state of participation in decision making with respect to PWDs in the study area.
- To determine the existing state of social inclusion of disabled persons in the area.
- To measure the association between participation in decision making and social inclusion of PWDs based on monthly family income, level of disability, and gender.

### 1.3. Research Questions

- What is the extent of social inclusion of PWDs with respect to their participation in cultural life and decision making?
- How do family type, gender, monthly family income, marital status and participation in decision making enhance the social inclusion of PWDs?

### 1.4. Purpose of the Study

People with disabilities possess a low socio-economic and political status the world over. However, the deprivation and exclusion of PWDs are especially high in low-income and developing countries like Pakistan. To enable the social inclusion of these people in mainstream society, various approaches have been adopted in different parts of the world. These approaches include charity, medical, social, and rights-based approaches. The rights-based approach, the most recent of all approaches, is based on the United Nations Convention on the Rights of Persons with Disabilities, which emphasizes the provision of the same rights to PWDs as enjoyed by an average person. Currently, almost one million people in Pakistan are disabled, and the number is expected to increase over time as the average age of the population increases. Sadly, most national developmental agendas have neglected the concept of disability in developmental goals, other than conceiving of PWDs as recipients of charity and welfare services. The fate of PWDs under past national developmental agendas was put in the hands of charitable organizations and professional experts, particularly doctors and rehabilitation and social care staff. This service delivery approach was changed to a rights-based approach in the late 1990s to enable PWDs to exercise their civil, political, social, economic, and cultural rights on an equal basis with others. The rights-based approach aims to promote, protect, and ensure the full and equal enjoyment of all human rights and fundamental freedoms by PWDs within the national legal framework. This approach tries to overcome exclusion and inequality at institutional, attitudinal, physical, legal, and communication levels. Therefore, the current study is designed to assess the state of participation in decision-making and the social inclusion of PWDs on quantitative grounds.

## 2. Study Methodology

This study, entitled “the influence of participation in decision making on the social inclusion of persons with disabilities: a case study of Khyber Pakhtunkhwa” was conducted in Malakand, Pakistan. To achieve the required results, the following procedure was adopted.

### 2.1. Universe of the Study

The study was conducted in district Malakand Khyber Pakhtunkhwa, Pakistan. The records available in the social welfare office at the district level (Malakand) show that the total number of PWDs is 8028. This includes physically disabled, crippled, and blind persons; however, data were only collected from those who were able to interview.

### 2.2. Research Design

The research study design was a cross-sectional, one-shot or status study, based on its time horizon. A cross-sectional study is the most appropriate design for understanding existing phenomena, problems, attitudes, perceptions or issues, and is performed by taking a cross-section of the population. This design gives an overall picture of the conditions at that time (i.e., at the time of the study). Such studies are cross-sectional with respect to both the time of exploration and the study population [32]. The focus of the study was to analyze the possible relationships between participation in decision-making and the social inclusion of persons with disabilities.

### 2.3. Unit of Observation and Unit of Analysis

The unit of analysis was the social unit, the characteristics of which were the primary focus of this research study [33]. Therefore, disabled persons, including those who were physically disabled and blind, were the unit of analysis for this study. In addition, the participation of PWDs in decision making at the individual, familial, and communal level and their state of social inclusion were considered the unit of observation.

### 2.4. Data and Data Collection Tools

For data collection, the following procedure was adopted.

#### 2.4.1. Operationalization of Variables

In this study, social inclusion was regarded as the availability of resources and opportunities to actively participate in all aspects of life and to enjoy standard life. Decision making refers to making decisions about medical treatment, employment, property, family, finances, and participation in social and cultural events.

#### 2.4.2. Instrumentation of Variables

The following procedure was opted to measure the association between the study variables, including one independent variable, namely participation in decision making, one dependent variable, i.e., social inclusion, and five background variables (gender, monthly family income, family type, marital status, and level of disability).

To measure the social inclusion of PWDs, A 13-items scale was developed, and positive responses on 7 or more items were considered to indicate the social inclusion of PWDs. The participation in decision making scale consisted of 9 items, and positive responses on 5 or more items were deemed to reflect active participation of PWDs in decision-making processes at personal, family, and communal levels.

#### 2.4.3. Data Collection Method and Tool

A well-taught interview schedule was developed and pre-tested for its relevance to the study’s objectives; the inconsistencies and ambiguities were corrected before actual data collection. The data were collected from male and female PWDs using interview methods while engaging male and female enumerators. The researchers and enumerators collected the data by visiting PWDs in their home, workplace, or any other place, as per their preference.

#### 2.4.4. Reliability Analysis

In the social sciences, 0.60 is the minimum level for reliability [34]. The reliability of the scales was measured using the following formula recommended by Cronbach [35]:

(1) $$\alpha =\frac{N\;.\bar{c}\;}{\left(\;\bar{v}+\left(N-1\right)\;.\;\bar{c}\right)}$$

The results of Cronbach’s alpha (α) test are given in Table 1.

#### 2.4.5. Indexation

Indexation is one of the tools used for assessing respondents’ perceptions about the study variables. There must be two or more statements in a variable for indexation purposes [36]. In this study, the variables were indexed and cross-tabulated to measure the association among variables at the multivariate level.

#### 2.4.6. Sample Size

A total of 8028 PWDs were registered with the government of Pakistan in the study area, constituting the population. The following formula, recommended by [37], was used to determine the study sample size.

(2) $$n=\frac{N\hat{p}\hat{q}Z^{2}}{\hat{p}\hat{q}Z^{2}+Ne^{2}-e^{2}}$$

A sample size of 488 PWDs was determined on the basis of the aforementioned formula.

### 2.5. Analytical Framework

The collected data were coded and entered into SPSS software version 24 for analysis. The data were analyzed at multivariate levels.

#### 2.5.1. Multivariate Analysis

Multivariate analysis was carried out to observe whether variation in the social inclusion of disabled persons caused by the participation in decision making could be explained by the respondent’s monthly family income, gender, or level of disability. Chi-square test and Kendall’s Tau-b test were used. The Chi-square [38] and Kendall’s [36] Tau-b formulas were employed as follows in the Equation (3) and (4) respectively:

(3) $$x^{2}=\overset{}{\underset{i}{\sum }}\frac{\left(O_{i-E_{i}}\right)}{E_{i}}\;$$

(4) $$\tau_{B}=\frac{n_{c}-n_{d}}{\sqrt{\left(n_{0}-n_{1}\right){\left(n_{0}-n_{2}\right)}^{}}}\;$$

#### 2.5.2. Logistic Regression Analysis

A logistic regression model was used to examine the relationship and association among group variables (marital status, family type, level of disability, monthly family income, and participation in decision making) and social inclusion of PWDs. The logistic regression model is most relevant when the respondents have two choices [39,40].

The formula for logistic regression recommended by [41], as given below, was used:

(5) $$Ln\left(\frac{P}{1-P}\right)=\beta_{0}+\beta_{1}X_{1}+\beta_{2}X_{2}+\ldots +\beta_{k}X_{k}$$

where *P* is the probability that Y = 1 given the values of the covariates, *β*_0_ is the intercept, *β*_1_, *β*_2_,..., *β_k_* are the coefficients of variables and *X* = (*X*_1_, *X*_2_... *X_k_*) is the set of explanatory variables.

To conduct a logistic regression analysis, Cronbach’s alpha and Chi-square tests were applied to measure the internal consistency and level of significance of the items under observation and were indexed. The indexed variables were measured on two-level scales and were defined as follows:
Family type0 nuclear family, 1 joint familyLiteracy status0 if illiterate, 1 literateMarital status0 if unmarried, 1 marriedMonthly family income0 if below PRs 16,500, 1 otherwiseParticipation in decision making0 if passive participation in decision making,
1 active participation in decision making

Omnibus test of the model coefficient was applied to measure the statistical significance of the full model against the constant-only model. The Wald test was used to predict the statistical significance of independent variables in predicting the dependent variable.

The logistic regression model used in this study to analyze the variables responsible for social inclusion of PWDs was as follows:

Y = a + b1X1 + b2X2 + b3X3 + b4X4 + b5X5(6)

Social inclusion of PWDs = a + b1 (family type) + b2 (literacy status) + b3 (marital status) + b4 (monthly family income) + b5 (participation in decision making).

### 2.6. Ethical Consideration/Participant Recruitment Information

Researchers in the social sciences have an ethical obligation to their colleagues, their study population, and the wider society. This research was approved by the ethical committee of Kohat University of Science and Technology (KUST), Kohat Pakistan. In this study, ethical issues were taken into consideration. The participants were assured of confidentiality and did not have to provide any personal information or identification if they did not wish to. The researchers ensured informed consent through study introduction and asked informants for voluntary participation. The researcher explained the study purpose to respondents to ensure that they understood what they were taking part in and how the data was used. Information from the target population was treated with the confidentiality that it deserved.

## 3. Results

### 3.1. *Participation in Decision Making*

Participation in decision making is an essential factor in measuring the social inclusion of individuals and groups, especially concerning PWDs. Each PWD can make decisions for themselves, or participate in family- and community-level decisions. Participation in decision making at these three levels creates a sense of ownership among PWDs concerning familial, communal, and societal activities. The personal decisions enable the PWDs to improve fundamental aspects of their lives, such as with respect to food, clothes and other basic needs. At the second level, the decisions help PWDs make choices regarding their health and education. The enabling environment in the first two tiers helps PWDs participate in the decision-making process regarding the welfare of the general masses. Therefore, the UNCRPD has made it mandatory to involve PWDs in all those decision-making processes that impact them. The respondents were asked some questions to measure their participation in decision-making processes, as shown in Table 2.

The results show that 63.1% of respondents refuted that they had choices about activities they want to do. Of the respondents, 52.9% were not entitled to participate in decision-making processes that affected them at home/work, and 56.1% of respondents did not question the events occurring around them at home/work. Likewise, 52.7% of respondents claimed that they were not entitled to participate in decision-making processes that affect their families. Their opinion being counted in family discussions was admitted by 51% of respondents, and 54.5% of respondents discussed uncertainties related to their life in the family. The results further show that entitlement to participate in decision-making processes that affect their community was refuted by 77% of respondents, while 90.3% of respondents stated that they were unable to participate in community decision making as much as they would like and that their opinion was not given due weight in overall decision-making processes (82%).

### 3.2. Multivariate

The multivariate results of the indexed independent variable (participation in decision making) and dependent variable (social inclusion) while controlling for gender, level of disability, and monthly family income of the PWDs are discussed in this section.

#### 3.2.1. Association between Participation in Decision Making and Social Inclusion (Controlling Gender)

The culture of patriarchy is maintained by observing gender-based distinctions in the division of labor during the socialization process. Children realize their gender-based norms, values, folkways, and taboos at an early age. Being enabled in decision making is also an essential aspect of socialization; however, females are trained in decision making regarding trivial issues, while males, to adopt their future role of household head, are engaged in critical decisions at the family and community level. On the same grounds, the opinions of male members have a greater weight than those of female members, and females are supposed to be more obedient. Therefore, the overall effect of enabling a decision-making environment is more positive and inclusionary for males than females. The results (Table 3) indicate that those disabled females describing good participation in decision making (44.2%) were more socially included, compared to 18% of those disabled females having poor participation in decision making. Similarly, for those males who have good participation in decision making, 64.7% were socially included, compared to 13.1% of those with poor participation in decision making. The influence of participation in decision making on social inclusion of PWDs in the context of the gender of the PWDS showed positive (T^b^ = 0.252) and significant association (*p* = 0.001) for females. Moreover, there was a significant (*p* = 0.000) and positive (T^b^ = 0.531) association for males. The entire table also indicates a highly significant and positive (*p* = 0.000, T^b^ = 0.458) association between social inclusion and participation in decision making for both genders. The results of Kendall’s Tau-b and Chi-square tests show a spurious relation between social inclusion and participation in decision making while controlling gender of the respondents. Furthermore, the results show that the participation in the decision making of PWDs enhances the social inclusion of male PWDs compared to female PWDs.

#### 3.2.2. Association between Social Inclusion and Participation in Decision Making While Controlling Monthly Family Income

The monthly family income plays an essential role in participating in the decision-making process at the personal, family, and community levels. The results (Table 4) indicate that those PWDs whose monthly family income is below PKR 16,500 and who have good participation in decision making (24.6%) were socially included in the mainstream society, compared to 4.1% of those with poor participation in decision making. Similarly, 81.4% of persons with disabilities with a monthly family income of PKR 16,500 and above actively participate in decision making, while the proportion of persons with a low degree of participation is 43.8%. The influence of participation in decision making on social inclusion of disabled persons based on monthly family income displayed a highly significant and positive (*p* = 0.000, T^b^ = 0.305) association for those with a monthly family income below PKR 16,500, and a highly significant and positive (*p* = 0.000, T^b^ = 0.391) association for PKR 16,500 and above. Furthermore, the entire table shows a highly significant (*p* = 0.000) and positive (T^b^ = 0.458) association between participation in decision making and social inclusion of PWDs for both monthly family income, i.e., below PKR 16,500 and 16,500 and above. The results of Kendall’s Tau-b (T^b^) and Chi-square significance values show a non-spurious relation between participation in decision making and social inclusion of PWDs when controlling for monthly family income. The results indicate that participation in decision making affects the social inclusion of PWDs universally, irrespective of their monthly family income.

#### 3.2.3. Association between Social Inclusion and Participation in Decision Making (Controlling the Level of Disability)

Enabling decision making is also an essential aspect of socialization and social inclusion. People with disabilities are dependent on other family members in decision making related to their daily needs at the personal and familial levels. The results (Table 5) show that among disabled persons with severe disability having good participation in decision making 58% were socially included, compared to 22.3% of those who with poor participation in decision making. Similarly, among those PWDs with moderate disability and good participation in decision making 62.9% were socially included, compared to 10.8% of those with poor participation in decision making. These results highlight a significant (*p* = 0.000) and positive (T^b^ = 0.366) influence of participation in decision making on social inclusion for PWDs with severe disability, and a positive (T^b^ = 0.538) and significant (*p* = 0.000) influence for moderately disabled persons. The entire table also depicts a significant (*p* = 0.000) and positive (T^b^ = 0.458) association between participation in decision making and social inclusion for both moderate and severe disability. The results of Kendall’s Tau-b values and Chi-square significance values indicate that the effects of participation in decision making and social inclusion of PWDs are spurious when controlling the level of disability. These results indicate that participation in decision making affects the social inclusion of moderately disabled PWDs more positively than those with severe disability.

### 3.3. Logistic Regression Analysis

The marital status, family type, monthly family income literacy status, level of disability, and participation in decision making of PWDs play an essential role in their social inclusion in mainstream society (Table 6). There were six independent variables, i.e., marital status, family type, level of disability, monthly family income, literacy status, and participation in decision making), that displayed a significant association in explaining variation in the social inclusion of PWDs (dependent variable) in the logistic regression model. All these variables were indexed; for measurement of variables, two-level scales, i.e., scores of 0 or 1, were used.

The results of the Chi-square omnibus test (306.114) and the significant (*p* = 0.000) values indicate that the overall model is statistically significant. Therefore, the grouping variables could well differentiate the deviations in social inclusion of disabled persons. Nagelkerke’s R Square (0.791) value shows that the group variable and prediction variables are strongly associated. Furthermore, the Cox and Snell R Square (0.466) and Nagelkerke’s R square (0.651) values indicated that the grouping variables explained 46% to 65% of the variation. The results of the Wald test for each variable support that all the grouping variables predict the social inclusion of disabled persons.

The results of the exponential-B value assisted in measuring the level of variation in the social inclusion of disabled persons under the influence of grouping variables. The (EXP (B) = 2.090) in the logistic regression model of this study indicated that PWDs from nuclear families are less likely to be socially included than those in joint families. These results further show that a change in literacy status from illiterate to literate can improve the chance of social inclusion of disabled persons by fourteen times (EXP (B) = 14.285). Moreover, the results revealed that between unmarried and married, marital status increases the chances of social inclusion of disabled persons by two times (EXP (B) = 2.395). In addition, the model shows that change in monthly family income from below PKR 16,500 to PKR 16,500 and above enhances the likelihood of social inclusion of PWDs by twelve times (EXP (B) = 12.480). Furthermore, the logistic regression model indicates that level of participation in decision making, from poor participation to good participation, increases the likelihood of social inclusion by almost three times (EXP (B) = 3.050) (Table 7).

The following is the equation for the logistic regression model:

Y = a + b1X1 + b2X2 + b3X3 + b4X4 + b5X5(7)

Social inclusion of PWDs = −5.000 + 0.737 (family type) + 0.874 (marital status) + 2.659 (literacy status) + 2.524 (monthly family income) + 1.15 (participation in decision making).

## 4. Discussion

The interplay between gender and disability makes the situation even harder for women with disabilities to execute their rights while facing oppressive and discriminatory obstacles, and they were less socially included than men in society [42]. It was noted by [26] that the active participation of women in decision making at all levels plays an important role in women’s empowerment and equality [23]. Women’s participation is vital in family decisions; however, in developing countries, women are discouraged from making decisions about themselves and their families [42,43]. Moreover, according to [44], women were found to be less confident in decision making to the extent that they could not make decisions on personal matters or independently spend money they had earned, probably, leading to their social exclusion [45].

The family income is important in reducing the economic deprivation of PWDs; however, participation in decision making requires additional input from families in order to socialize their members. Thus, disability increases vulnerability to low participation in social activities, especially when disability is stigmatized. PWDs face numerous problems like health complications and poverty, which become stigmatized in society [28]. This stigmatization can be overcome through economic independence through creating employment opportunities [7,46]. Economic stability cannot be the sole factor in removing stigma; instead, PWDs should be equipped with the strengths to be able to access their requirements effectively and to be able to overcome challenges in their employment [7,47]. Economic status is a significant contributor to securing high levels of prestige and a prosperous life. Therefore, PWDs with a high family status have more chances of social inclusion than those from low-income families. The ability to avail themselves of the opportunities of quality education, health, mobility and transport, participation in cultural life, and decision making requires financial resources; however, most PWDs face barriers when accessing services due to their low income and unemployment [48,49].

Family is the most important agent of socialization, ultimately providing productive members to society [50]. The proper socialization of individuals at the family level has lifelong effects on their lives and quality of life, resulting in the social inclusion of PWDs [51]. There are fewer chances for PWDs in nuclear families to be productive members, leading them to be considered a burden on others; the unsupportive attitudes of family members decrease the chances of their social inclusion [52,53]. The care of children with disabilities in nuclear families is considered a burden and a source of stress for the whole family; however, joint families can easily handle all the problems of disabled children [48,52,54].

Education is an essential agent of socialization after the family, and enables individuals to contribute to society through their educational knowledge and skills. The maximum inputs and efforts are required for the education of PWDs is the reason for their low literacy that ultimately leads to low participation of PWDs in decision making as well as social exclusion [47,53,55]. Peers and friends are a significant source of support and motivation for disabled children [56]. This kind of support and motivation can improve the literacy status of PWDs as well as their social inclusion [57]. Therefore, education is the most substantial element in reducing the barriers that hinder social inclusion [48].

Marriage is established as an institution that satisfies multiple basic human needs, e.g., the fulfilment of sexual desire for both sexes, and ultimately the production of children. PWDs face difficulty marrying someone, starting a family, and playing the roles of parents due to various barriers in the cultural setup. Therefore, most PWDs remain unmarried throughout their lives and are socially excluded [58,59,60].

People with severe disabilities are dependent on other family members in decision making related to their daily needs at the personal and familial levels. On the other hand, people with moderate disabilities make decisions regarding their daily routine and are less dependent on others. They have more chances to interact with the wider society and can make decisions about their personal, familial, and communal matters. Therefore, the overall effect of enabling a decision-making environment for PWDs with moderate disabilities is more positive and inclusionary than PWDs with severe disabilities. Participation in decision making is a foundation of good feelings, and is an essential source of physical, psychological, and social well-being, as well as quality of life, for PWDs [61]. It also enhances their participation in all aspects of their lives, ultimately leading to the development of a sense of independence and inclusion [17]. Lack of access to decision making for PWDs [18] has dire consequences on personal well-being and survival [20,62,63]. Ref. [50] found that PWDs with severe disability believed that they could not speak on their own behalf, and therefore were not competent to make their own decisions.

Participation in decision making is a fundamental human right for all, including PWDs. Active participation in decision making at the personal, familial, and communal levels gives hope to PWDs that they can be productive members of society, and can consider themselves as a value part of society, compelling their social inclusion in mainstream society [64,65].

## 5. Conclusions

This study was carried out to explore different factors responsible for the social inclusion of PWDs. These factors were measured on the basis of participation in decision making, gender, family type, level of disability, marital status, and monthly family income. The effects of these independent variables were noticed in the dependent variable (social inclusion of PWDs). Disability is widespread, especially in young adults and adults of both genders and all family types. Moreover, PWDs were illiterate or had poor education, and belonged to low-income families of large size. The combination of disability and poverty had drastic consequences for the economic and familial life of PWDs in terms of poor participation in decision making, unemployment, and remaining unmarried. They were found to be less interested in making personal-, familial-, and community-level decisions. Their opinions were mainly overlooked in such decisions, ultimately leading to their low level of social inclusion in mainstream society. The social inclusion of PWDs varied according to their socio-economic status, literacy status, marital status, gender, and level of disability. This study found that PWDs were unable to actively participate in the decision-making process at the personal, family, and community level; however, those who actively participated in decision-making processes, were moderately disabled, were from a joint family system, were male, and were married were more socially included in mainstream society. This study suggests that the social inclusion of PWDs can be improved by favoring marriages, joint family systems, education, and improved access to decision-making processes. In addition, it is important to ensure participation of PWDs in decision-making processes in family, community, school and other relevant institutions in order to ensure that the needs of PWDs are properly integrated at all these levels, and that their needs are satisfactorily provided for at each institutional level.

### Limitations of the Study

This research study was limited to government-registered PWDs only. It is pertinent to mention that only a handful PWDs are registered with the social welfare department. The social workers fear that the social welfare department lacks actual population statistics in the district. Furthermore, the number of female registered PWDs was negligible for cultural reasons. Due to financial and time constraints, the researchers were not able to reach extremely distantly located geographical areas.

## Figures and Tables

**Table 1:** Cronbach’s alpha (α) results/values of scales.

Variable	Cronbach’s Alpha (α) Value
Social inclusion of PWDs	0.815
Participation in decision making	0.805

**Table 2:** Frequency distribution and proportion of respondents showing their participation in decision making.

Statement	Yes	No	Total
You have choices about activities you want to do	180 (36.9)	308 (63.1)	488 (100)
You are entitled to participate in decision making which affects you at home/work	230 (47.1)	58 (52.9)	488 (100)
You question the events occurring around you at home/work	214 (43.9)	274 (56.1)	488 (100)
You are entitled to participate in decision making which affects your family	231 (47.3)	257 (52.7)	488 (100)
Your opinion is counted in family discussions	249 (51.0)	239 (49.0)	488 (100)
You discuss uncertainties related to your life in family	266 (54.5)	222 (45.5)	488 (100)
You are entitled to participate in decision making which affects your community?	112 (23.0)	376 (77.0)	488 (100)
You participate in community decision making as much as you would have liked?	47 (9.7)	441 (90.3)	488 (100)
Your opinion is given due weight in the overall decision-making process	88 (18.0)	400 (82.0)	488 (100)

**Table 3:** Social inclusion of PWDs and participation in decision making while controlling gender.

Gender	Participation in Decision Making	Social Exclusion	Social Inclusion	Total	Statistics χ^2^ (*p*-Value) T^b^	Level of Significance for Entire Table
Female	Poor participation in decision making	132 (82.0)	29 (18.0)	161 (100)	χ^2^ = 12.921 *p* = 0.001 T^b^ = 0.252	χ^2^ = 102.189 *p* = 0.000 T^b^ = 0.458
	Good participation in decision making	24 (55.8)	19 (44.2)	43 (100)		
	Total	156 (76.5)	48 (23.5)	204 (100)		
Male	Poor participation in decision making	126 (86.9)	19 (13.1)	145 (100)	χ^2^ = 80.037 *p* = 0.000 T^b^ = 0.531	
	Good participation in decision making	49 (35.3)	90 (64.7)	139 (100)		
	Total	175 (61.6)	109 (38.4)	284 (100)		

**Table 4:** Social inclusion of PWDs and participation in decision making while controlling monthly family income.

Monthly Family Income	Participation in Decision Making	Social Exclusion	Social Inclusion	Total	Statistics χ^2^ (*p*-Value) T^b^	Level of Significance for Entire Table
below than PRs 16,500	Poor participation in decision making	208 (95.9)	9 (4.1)	217 (100)	χ^2^ = 26.596 *p* = 0.000 T^b^ = 0.305	χ^2^ = 102.189 *p* = 0.000 T^b^ = 0.458
	Good participation in decision making	52 (75.4)	17 (24.6)	69 (100)		
	Total	260 (90.9)	26 (9.1)	286 (100)		
PRs 16,500 and above	Poor participation in decision making	50 (56.2)	39 (43.8)	89 (100)	χ^2^ = 30.872 *p* = 0.000 T^b^ = 0.391	
	Good participation in decision making	21 (18.6)	92 (81.4)	113 (100)		
	Total	71 (35.1)	131 (64.9)	202 (100)		

**Table 5:** Social inclusion of PWDs and participation in decision making while controlling the level of disability.

Level of Disability	Participation in Decision Making	Social Exclusion	Social Inclusion	Total	Statistics χ^2^ (p-Value) Tb	Level of Significance for Entire Table
Severe disability	Meager participation in decision making	101 (77.7)	29 (22.3)	130 (100)	χ^2^ = 32.330 *p* = 0.000 T^b^ = 0.366	χ^2^ = 102.189 *p* = 0.000 T^b^ = 0.458
	Good participation in decision making	47 (42.0)	65 (58.0)	112 (100)		
	Total	148 (61.2)	94 (38.8)	242 (100)		
Moderate disability	Poor participation in decision making	157 (89.2)	19 (10.8)	176 (100)	χ^2^ = 71.251 *p* = 0.000 T^b^ = 0.538	
	Good participation in decision making	26 (37.1)	44 (62.9)	70 (28.5)		
	Total	183 (100)	63 (100)	246 (100)		

**Table 6:** Descriptive statistics.

Variable	Mean	Std. Deviation	Variance
Family Type	0.756	0.430	0.185
Gender of the Respondents	0.582	0.493	0.244
Marital Status	0.688	0.463	0.215
Monthly family income	0.414	0.493	0.243
Level of Disability	0.504	0.500	0.250
Participation in Decision Making	0.370	0.484	0.234
Social Inclusion of PWDs	0.643	0.568	0.269

**Table 7:** Influence of family type, literacy status, marital status, monthly family income and participation of decision making on social inclusion of PWDs.

Independent Variables	Un-Standardized Coefficient		EXP B	Wald Test Value	Sig.	Omnibus Test	Model Summary		
	B	Std. Error				Chi-Square	Sig.	Cox and Snell R.Sqaure	Nagelkerke’s R.Sqaure
Family type	0.737	0.358	2.090	4.234	0.040	306.114	0.000	0.466	0.651
Literacy status	2.659	0.336	14.285	62.812	0.000				
Marital Status	0.874	0.347	2.395	6.355	0.012				
Monthlyfamily income	2.524	0.304	12.480	69.003	0.000				
Participation in Decision Making	1.115	0.329	3.050	11.476	0.001				
Constant	−5.000	0.547	0.007	83.686	0.000				

## Data Availability

The data presented in this study are available on request. The data are not publicly available due to privacy.
